# Equipping non-radiologist with basic point of care ultrasound skills- it can save lives!

**DOI:** 10.1186/2036-7902-7-S1-A4

**Published:** 2015-03-09

**Authors:** YK Liew

**Affiliations:** Accident and Emergency Department, Hospital Kapit, Kapit, Malaysia

**Keywords:** Referral Centre, Germ Cell Tumour, Pericardial Effusion, Cardiac Tamponade, Pericardial Fluid

## Background

The author serves in a resource limited and logistically challenging rural district hospital in the state of Sarawak, Malaysia. Transfer of patients requiring higher level of care to our nearest referral centre can only be done with 3 hours of boat ride or 1 hour by helicopter. Subspecialty centres, like our cardiac centres, are located even further away and can only be reached directly by helicopter service. We recently encountered a case of a patient admitted into our ward diagnosed to have an acute onset of cardiac tamponade via point of care ultrasound (POCUS) performed by the author, a non-radiologist. Including the author, only 2 out of 10 medical officers in our hospital receive formal training in delivering POCUS. This case report serves to emphasise on the training, utility and benefits of POCUS to frontliners serving in austere working environment.

## Case presentation

A 19 year old gentleman was initially treated for pulmonary meliodosis at our setting due to the endemicity of the disease and suggestive clinical findings. However, 2 weeks into treatment, the patient persistently exhibit high spiking temperature and was clinically tachypneoic and tachycardic with a normal blood pressure. Bedside transthoracic scan at that time shows pleural effusion with hepatisation of right lung with no pericardial fluid. A repeat transthoracic scan the next day reveals a newly developed pericardial effusion with scalloping of the right ventricular free wall suggestive of cardiac tamponade. The patient was then transferred directly to the cardiac centre via helicopter service for emergency pericardiocentesis. He was later found to have non seminamatous germ cell tumour and appropriate treatment was instituted thereafter.

## Discussion

Cardiac tamponade is not a commonly encountered occurrence. However, missing out on this finding would have led to a devastating sequelae. Classic clinical findings only occur in minority of patients and may be unreliable as it can be due to other pathological processes. [1]

The utility of POCUS has helped enhance our clinical assessment, facilitate the diagnosis of cardiac tamponade and enabled us to arrange for a direct helicopter transfer to our cardiac referral centre, bypassing our intermediate referral centre. This has indeed saved precious time!

The role of point of care ultrasonography is identified in disciplines ranging from anaesthesiology to obstretics and gynaecology to even orthopaedic surgery [2]. However practical ultrasonography is not part of many medical curriculum and is still widely regarded as the domain of the radiologist.[3]

Centres which are less equipped and with no radiologist support, like ours, stand to greatly benefit with the availability of ultrasound machine.[4–6]

Formal training and teaching of POCUS in the curriculum will greatly help to enhance accurate diagnosis especially in critical care setting.[7]

## Informed consent

The study was conducted in accordance with the ethical standards dictated by applicable law. Informed consent was obtained from each owner to enrolment in the study and to the inclusion in this article of information that could potentially lead to their identification.
